# Simultaneous Quantitation of Cationic and Non-ionic Contrast Agents in Articular Cartilage Using Synchrotron MicroCT Imaging

**DOI:** 10.1038/s41598-019-43276-6

**Published:** 2019-05-08

**Authors:** Annina E. A. Saukko, Mikael J. Turunen, Miitu K. M. Honkanen, Goran Lovric, Virpi Tiitu, Juuso T. J. Honkanen, Mark W. Grinstaff, Jukka S. Jurvelin, Juha Töyräs

**Affiliations:** 1 Department of Applied Physics, University of Eastern Finland, POB 1627, FI-70211 Kuopio, Finland; 2 Diagnostic Imaging Center, Kuopio University Hospital, POB 100, FI-70029 Kuopio, Finland; 3 Centre d’Imagerie BioMédicale, École Polytechnique Fédérale de Lausanne, Lausanne, 1015 Switzerland; 4 Swiss Light Source, Paul Scherrer Institute, 5234 Villigen, Switzerland; 5 Institute of Biomedicine, Anatomy, University of Eastern Finland, POB 1627, FI-70211 Kuopio, Finland; 6 Center of Oncology, Kuopio University Hospital, POB 100, FI-70029 Kuopio, Finland; 7 Departments of Biomedical Engineering, Chemistry, and Medicine, Boston University, 590 Commonwealth Ave., Boston, MA 02215 USA; 8 School of Information Technology and Electrical Engineering, The University of Queensland, Brisbane, QLD 4072 Australia

**Keywords:** Biological physics, Imaging techniques

## Abstract

Early diagnosis of acute cartilage injuries enables monitoring of disease progression and improved treatment option planning to prevent post-traumatic osteoarthritis. In contrast-enhanced computed tomography (CECT), the changes in cationic agent diffusion within the tissue reflect cartilage degeneration. The diffusion in degenerated cartilage depends on proteoglycan (PG) content and water content, but each having an opposite effect on diffusion, thus compromising the diagnostic sensitivity. To overcome this limitation, we propose the simultaneous imaging of cationic (sensitive to PG and water contents) and non-ionic (sensitive to water content) agents. In this study, quantitative dual-energy CT (QDECT) imaging of two agents is reported for the first time at clinically feasible imaging time points. Furthermore, this is the first time synchrotron microCT with monochromatic X-rays is employed in cartilage CECT. Imaging was conducted at 1 and 2 h post contrast agent immersion. Intact, PG-depleted, and mechanically injured + PG-depleted cartilage samples (*n* = 33) were imaged in a mixture of cationic (iodine-based CA4+) and non-ionic (gadolinium-based gadoteridol) agents. Concurrent evaluation of CA4+ and gadoteridol partitions in cartilage is accomplished using QDECT. Subsequent normalization of the CA4+ partition with that of the gadoteridol affords CA4+ attenuations that significantly correlate with PG content – a key marker of OA.

## Introduction

Post-traumatic osteoarthritis (PTOA) typically develops after acute traumas, such as sport injuries or sudden impacts, for example, during vehicle accidents or falls. Cartilage damage, subchondral bone contusions, torn ligaments or meniscal tears are characteristic tissue features after acute joint injuries. Furthermore, abnormal joint function after injuries may create excessive tissue stresses and strains that lead to progressive loss of joint cartilage and subsequent osteoarthritis (OA). Recent findings suggest that the early detection of original tissue injury enables surgical and pharmaceutical interventions to prevent or slow down the development of PTOA^1,2^. In this context, techniques which enable sensitive detection of lesions and early post-traumatic degeneration of surrounding cartilage tissue are of interest and needed. Magnetic resonance imaging (MRI) is used to identify cartilage tissue properties, such as PG content, collagen orientation, and water content^3,4^. As MRI exhibits excellent soft tissue contrast, it is an important clinical tool in cartilage imaging. However, MRI provides relatively low spatial resolution of the tissue and requires relatively long *in vivo* image acquisition times^4,5^.

As an alternative, contrast-enhanced computed tomography (CECT) provides high-resolution images of cartilage with short scan times at approximately half the cost of MRI. CECT also allows the early detection of tissue changes after an acute injury. Although CECT has been recently introduced in clinics, it is not yet widely used^6–10^. In CECT, contrast agents enhance the visualization of the cartilage tissue, as well as the cartilage-bone and synovial fluid-cartilage interfaces. Although the natural difference in the X-ray absorption at the synovial fluid-cartilage interfaces is nearly non-existent, CECT enables separation of these tissues and their boundaries. Further, degenerative cartilage changes are detected by examining the uptake and partitioning of mobile, ionic contrast agents in tissue^11–16^. The early degenerative changes (i.e., decrease in cartilage fixed charge density (FCD) via loss of PGs, increase in cartilage water content, and disruption of superficial collagen network^17,18^) alter the uptake of the contrast agent into cartilage tissue^19^.

Typically, CECT relies on two subsequent CT scans conducted immediately and at delayed time point, e.g. approximately 45 minutes after the intra-articular injection of the contrast agent^6–8^. The first scan (arthrography) is needed for identification of articulating surfaces and surface lesions while the second scan (delayed arthrography) allows the evaluation of internal integrity of the cartilage and assessment of PTOA. A previous study showed that the optimal time point for delayed imaging is 30–60 minutes after administration of the anionic contrast agent in order to achieve maximum contrast agent concentration in cartilage^6^.

Anionic, iodine-based molecules are the most commonly used contrast agents in knee CECT^7,8,16^. Owing to their negative charge, anionic contrast agents distribute by diffusion into cartilage, inversely proportionally to the spatial distribution of the negatively charged PGs^20,21^. Recent findings show that cationic contrast agents are more sensitive to detect changes in PG content at diffusion equilibrium than the conventional anionic contrast agents^22–24^. However, a limitation with cationic contrast agents is that their diffusion into degenerated cartilage tissue is controlled by two factors with opposite effects. The loss of PGs decreases the uptake (less attraction) while increased water content and reduced steric hindrance enhance the diffusion of cationic agents into cartilage, impairing diagnostic sensitivity of CECT especially at early imaging time points.

A novel approach to improve the capability of CECT is to apply a quantitative dual-energy CT (QDECT) technique^25^. This technique employs the use of a mixture of two contrast agents: 1) a cationic, iodinated contrast agent (CA4+)^14,22^; and 2) a non-ionic, gadolinium-based contrast agent (gadoteridol). In the dual contrast agent approach, CA4+ possesses a high affinity for the negative charge of PGs and distributes into cartilage proportionally to PG content. Gadoteridol, on the other hand, distributes into cartilage in relation to water content and steric hindrance of the tissue (i.e., physical diffusion barrier caused by collagen network architecture and PGs in the matrix), and is unaffected by the negative charge of PGs. Thus, the diagnostic value of CA4+ in clinical applications with a typical diffusion time frame of 30–60 minutes may be improved by normalizing the uptake of CA4+ with that of gadoteridol. Here, the normalization is achieved by dividing the CA4+ uptake in cartilage with that of gadoteridol.

With QDECT, the uptake of CA4+ and gadoteridol within cartilage are measured simultaneously by imaging with two different X-ray energies which represents a significant advantage over conventional CECT technique. Specifically, QDECT relies on element specific absorption *k*-edge energies of 33.2 and 50.3 keV for iodine and gadolinium, respectively. In this context, the QDECT technique allows the simultaneous quantification of interstitial water and PG contents in articular cartilage based on CA4+ and gadoteridol distributions within tissue, thus enabling better diagnostic sensitivity for tissue degradation and osteoarthritic stage. The current technique also enables early detection of acute cartilage injuries by simultaneously providing information on the composition of cartilage, which is not currently possible with the standard techniques such as CT and MRI. Thus, QDECT provides an important alternative for MRI and CT imaging in cases where more specific information is needed in order to select the best treatment option after acute cartilage injury.

One of the limitations of conventional microCT and clinical CT scanners is that they lack imaging with monochromatic energies and this inevitably leads to beam hardening artifacts. Beam hardening reduces the accuracy of current CT technique. In beam hardening, the average photon energy in the X-ray beam is increased due to selective attenuation of lower energy photons, shifting the energy distribution of the X-ray beam away from the *k*-edge of iodine and towards that of gadolinium, resulting in a higher proportion of the higher energies exceeding the *k*-edge of gadolinium. This shift reduces the attenuation caused by iodine, while minimally affecting the attenuation by gadolinium^26^. In addition, when high resolution is required, conventional microCT instruments also require long imaging times. This limitation hinders the diagnostic sensitivity of the QDECT techniques due to continued diffusion of the contrast agents.

In the present study, synchrotron microCT is used as it provides imaging with monochromatic energies contemporally offering fast tomographic imaging with high resolution^27^. Specifically, QDECT is evaluated using intact, PG-depleted, and mechanically injured + PG-depleted samples. The first aim addresses the hypothesis that the uptake of cationic, iodine-based and non-ionic, gadolinium-based contrast agents can be determined simultaneously at clinically feasible diffusion time points (at 1 and 2 h after immersion). Further, we hypothesize that the differences between the intact, PG-depleted, and mechanically injured + PG-depleted samples can be quantitatively determined. The second aim addresses the hypothesis that the use of monochromatic X-ray energies provides sensitive QDECT diagnostics without the limitations commonly encountered with conventional CT scanners. We characterize the effect of mechanical impact, known to reduce steric hindrance on contrast agent diffusion^28^. As the first study to investigate simultaneously the diffusion of two different contrast agents into cartilage using separate monochromatic X-ray energies and the fact that dual-energy CT scanners are becoming more common in hospitals, these results provide a basis for a future quantitative diagnostic method for cartilage health and injury.

## Materials and Methods

### Sample preparation

Intact, skeletally mature bovine knee joints (*N* = 11) were obtained and patellae were carefully dissected. Three adjacent osteochondral plugs (*n* = 33, *d* = 4 mm) were extracted from the upper lateral quadrant of each patellae. The samples were kept moist by using phosphate buffered saline (PBS) during the extraction process. The number of samples was selected based on our previous papers^29–33^ and the number was maximized to capitalize on the beamtime allocated to us by Paul Scherrer Institute (PSI). No ethical permission or approval for the experimental protocol and tissue collection were needed as the samples were obtained from a local grocery store (Prisma, Kuopio, Finland). After detaching the samples, the adjacent osteochondral plugs from the same patellae were divided into three groups: 1) intact, 2) PG-depleted, and 3) mechanically injured + PG-depleted. Preparation of PG depleted osteochondral plugs were prepared by enzymatic (trypsin) degradation. Degradation was performed by immersing the samples in PBS supplemented with trypsin (0.5 mg/ml, Sigma-Aldrich, MO, USA) for 15 h at 37.5 °C in an incubator^34^. After 15 h, the process was suppressed and the trypsin was washed out by immersing the samples in PBS for 2 h at 7 °C. With enzymatic degradation, early-stage OA, which is characterized by loss of PGs, is simulated using these enzymatically degraded plugs^35,36^.

After the enzymatic degradation, the samples from the group 3 were injured mechanically using a custom-made drop tower. Mechanical impact on cartilage is known to reduce steric hindrance of articular cartilage and thus increase the uptake of contrast agent^28,32^. To mechanically damage the cartilage, a 500 g stainless steel impactor with a polished face was dropped on the sample from the height of 6.7 cm. The magnitude of impact was *P* = 0.387 kgm/s as used in previous studies^29,32^. The impactor was lifted immediately after the impact to prevent any cartilage creep deformation, after which the samples were kept immersed in PBS for 1 h to allow recovery from the impact. The procedure creates minor cracks on the surface of the cartilage^29,32^. Subsequently, the samples were immersed in PBS and frozen at −20 °C until thawing prior to synchrotron microCT imaging. All experimental protocols were carried out in accordance with the guidelines and regulations.

### Synchrotron microCT imaging

The dual contrast method was examined by utilizing a third-generation synchrotron-based X-ray source (X02DA TOMCAT beamline of the Swiss Light Source, PSI, Villigen, Switzerland)^37^. With this synchrotron-based microCT technique, the X-ray beams are produced by a 2.9 T super-bending magnet on a 2.4 GeV storage ring (operated in top-up mode and 400 mA ring current). Monochromatic X-ray energies with an approximate energy bandwidth of 2–3% are tuned by using a double-multilayer monochromator (DMM) and ensure maximum difference between CA4+ and gadoteridol X-ray attenuation during imaging, thereby allowing high accuracy detection of contrast agent partitions within cartilage. The osteochondral samples, contrast agent calibration series, and a set of contrast agent mixtures described in more detail later were imaged by coupling a 1:1 magnifying visible light optics with a 300 µm thick scintillator (LuAg) in combination with a scientific CMOS detector (pco.Edge 4.2). Two X-ray energies (25 keV and 37 keV) with a field of view (FOV) of 17.07 mm × 17.07 mm × 2.75 mm and a minimal time difference (~5 min 20 s) between the scans were utilized with an effective pixel size of 6.5 × 6.5 µm^2^. The samples were placed at a distance of 26.3 m away from the X-ray source while the detector was placed downstream as close as possible (<5 mm) to the samples in order to avoid edge-enhancement artifacts caused by Fresnel diffraction. Thus, only the bordering pixels of the air-to-sample interface were affected by Fresnel diffraction and these pixels were not used for the subsequent analysis^38,39^. The acquisition times for each tomographic scan were approximately 128 seconds. The reconstruction was performed using a highly optimized algorithm based on Fourier methods^40^. Off-beam sample alignment was used to minimize the radiation exposure to the samples during the alignment procedures^41^. The single-projection entrance doses (i.e., skin doses), with cartilage tissue modelled as water, were measured with a calibrated passivated implanted planar silicon (PIPS) diode32 and yielded 2.7 Gy and 1.1 Gy for the two energies (25 keV and 37 keV), respectively.

First, the calibration series of solutions with varying iodine (CA4+) and gadolinium (gadoteridol) concentrations in distilled water were imaged using both energies to determine the mass attenuation coefficients of the contrast agent compounds. In the solutions, the iodine concentration was 0.5, 1, 1.5, 2, 2.5, 3, 5, 10, 15, 20 mg I/mL and the gadolinium concentration 1, 2, 3, 4, 5, 7.5, 10, 12.5, 15 mg Gd/mL. In order to validate this method, four different mixtures of iodine- and gadolinium-based contrast agents in distilled water were imaged after the calibration series. The compositions of the mixtures were 1) 20 mg I/mL and 5 mg Gd/mL, 2) 10 mg I/mL and 10 mg Gd/mL, 3) 5 mg I/mL and 20 mg Gd/mL, and 4) 3 mg I/mL and 3 mg Gd/mL.

Prior to imaging, the side and the bottom of each osteochondral plug were sealed carefully using cyanoacrylate (Loctite, Henkel Norden AB, Dusseldorf, Germany). Thereby, the contrast agent diffusion was possible only through the articulating surface. To determine the X-ray attenuation profile in native cartilage the osteochondral samples were imaged in air with both energies before the contrast agent immersion. Then, the samples were immersed in isotonic (~308 mOsm/kg) mixture of iodinated, cationic contrast agent (CA4+, *q* =  + 4, *M* = 1499.87 g/mol) and gadolinium-based, electrically neutral contrast agent (gadoteridol, *q* = 0, *M* = 558.69 g/mol, ProHance^TM^, Bracco Diagnostic Inc., Monroe Twp., NJ, USA) diluted in PBS. In the mixture, the iodine and gadolinium concentrations were 5 mg I/ml and 10 mg Gd/ml. Further, proteolytic inhibitors [5 mM ethylenediaminetetraacetic acid (EDTA, VWR International, France) and 5 mM benzamidine hydrochloride hydrate (Sigma-Aldrich Inc., St Louis, MO, USA)] were added to prevent general protein degradation in tissue. The samples were kept immersed in 3 ml of the contrast agent mixture for 2 h at 7 °C and imaged in air at 1 and 2 h time points with both energies. After the microCT imaging, the samples were immersed in a 60 ml bath of PBS for 6 h at 7 °C to wash out the contrast agent from the cartilage tissue and subsequently frozen until histological analysis.

### Image analysis

The concentrations of iodine and gadolinium in the mixtures and partitions within cartilage can be solved using Beer-Lambert law and Bragg’s rule^42^:

1 $${I}_{E}={I}_{0,E}{e}^{-{\alpha }_{E}}$$

2 $${\alpha }_{E}={\mu }_{{\rm{I}},E}{C}_{{\rm{I}}}+\,{\mu }_{{\rm{G}}{\rm{d}},E}{C}_{{\rm{G}}{\rm{d}}},$$

where *I*_*E*_ and *I*_0,*E*_ are the intensities of the transmitted and incident X-ray beams of energy *E*, *α*_*E*_ the attenuation coefficient, *μ*_I,*E*_ and *μ*_Gd,*E*_ the mass attenuation coefficients, and *C*_I_ and *C*_Gd_ the concentrations of iodine (I) and gadolinium (Gd). Thus, the concentrations *C*_I_ and *C*_Gd_ can be solved from the following equations (in the case X-ray energies 25 keV and 37 keV are used):

3 $${C}_{{\rm{I}}}=\frac{{\alpha }_{37{\rm{k}}{\rm{e}}{\rm{V}}}{\mu }_{{\rm{G}}{\rm{d}},25{\rm{k}}{\rm{e}}{\rm{V}}}-{\alpha }_{25{\rm{k}}{\rm{e}}{\rm{V}}}{\mu }_{{\rm{G}}{\rm{d}},37{\rm{k}}{\rm{e}}{\rm{V}}}}{{\mu }_{{\rm{I}},37{\rm{k}}{\rm{e}}{\rm{V}}}{\mu }_{{\rm{G}}{\rm{d}},25{\rm{k}}{\rm{e}}{\rm{V}}}-{\mu }_{{\rm{I}},25{\rm{k}}{\rm{e}}{\rm{V}}}{\mu }_{{\rm{G}}{\rm{d}},37{\rm{k}}{\rm{e}}{\rm{V}}}}$$

4 $${C}_{{\rm{G}}{\rm{d}}}=\frac{{\alpha }_{25{\rm{k}}{\rm{e}}{\rm{V}}}{\mu }_{{\rm{I}},37{\rm{k}}{\rm{e}}{\rm{V}}}-{\alpha }_{37{\rm{k}}{\rm{e}}{\rm{V}}}{\mu }_{{\rm{I}},25{\rm{k}}{\rm{e}}{\rm{V}}}}{{\mu }_{{\rm{I}},37{\rm{k}}{\rm{e}}{\rm{V}}}{\mu }_{{\rm{G}}{\rm{d}},25{\rm{k}}{\rm{e}}{\rm{V}}}-{\mu }_{{\rm{I}},25{\rm{k}}{\rm{e}}{\rm{V}}}{\mu }_{{\rm{G}}{\rm{d}},37{\rm{k}}{\rm{e}}{\rm{V}}}}.$$

The articulating surface and bone-cartilage interface were first delineated manually from the CT image stacks using a segmentation software (Seg3D v2.4, The University of Utah, Salt Lake City, UT, USA). Then, the CA4+ and gadoteridol partition profiles through the cartilage layer were calculated using a custom-made MATLAB (R2014a, MathWorks, Inc., USA) script as described in more detail in Saukko *et al*.^29^. The volume of interest (VOI) was selected to be 2275 μm × 2275 μm × cartilage thickness and was delineated from the CT image stack to closely preserve the initial volume of cartilage. Next, the mean vertical X-ray attenuation profiles from the subchondral bone through to the articular surface were obtained by averaging the pixels in the horizontal direction in the VOI for native cartilage as well as for the samples imaged at the 1 h and 2 h time points. From the X-ray attenuation profiles, the concentrations of CA4+ (iodine, *C*_I_) and gadoteridol (gadolinium, *C*_Gd_) in cartilage were calculated (Eqs 3 and 4). Equations 3 and 4 require data on attenuation caused by iodine and gadolinium. Consequently, the attenuation as a result of cartilage tissue and water present in the VOI was eliminated from the 1 h and 2 h X-ray attenuation profiles. This was accomplished by subtracting the X-ray attenuation profile of native cartilage tissue from the attenuation profiles recorded on each time point, after which Eqs 2 and 3 were used to calculate the concentrations of iodine and gadolinium in the cartilage.

Partitions of CA4+ and gadoteridol were determined by dividing the obtained contrast agent concentration inside the cartilage with the contrast agent concentration in the immersion bath. Finally, the CA4+ partition was normalized by dividing the CA4+ partition in cartilage with the gadoteridol partition. This was performed to minimize the effects on uptake of cationic contrast agent, as caused by a difference in water content and steric hindrance. The normalization was assumed to improve the diagnostic sensitivity of cationic contrast agent for cartilage PG content. Further, CA4+ and gadoteridol concentration maps of the cartilage were calculated by averaging the image slices in the VOI in the horizontal direction. After obtaining the average X-ray attenuation in the cartilage tissue and water from the baseline image stack, their effect was removed by subtracting the baseline image stack from the 1 and 2 h images similarly as was done when calculating the X-ray attenuation profiles. Then, the CA4+ and gadoteridol distributions were calculated for each pixel in the image (Eqs 3 and 4). Finally, the CA4+ and gadoteridol distributions within the cartilage samples were averaged to obtain the average distributions of the contrast agents.

### Histological analysis

After synchrotron microCT imaging, the frozen samples were thawed and cut in half. The first half was fixed in 10% formalin and then dehydrated in ascending series of ethanol for the reference histological analysis. After dehydration, the samples were decalcified in EDTA and embedded in paraffin in order to cut 3 µm thick sections. Then, the paraffin was removed, and Safranin-O staining was performed to study the spatial FCD (i.e. PG) distribution in the cartilage. After staining, quantitative digital densitometry measurements were conducted to study the optical density (OD, e.g., PG distribution) in cartilage using a light microscope (Nikon Microphot-FXA, Nikon Co., Japan) equipped with a monochromatic light source and a 12-bit CCD camera (ORCA-ER, Hamamatsu Photonics K.K., Japan). System was calibrated using neutral density filters (Schott, Germany) covering OD range from 0 to 2.6. Histological images of Safranin-O stained sections were also imaged with a light microscope (Leica MZ75, Leica Microsystems Ltd., Switzerland) fitted with a CCD camera (Nikon digital sight DS-Fi2, Nikon Co., Japan). The depth-wise PG content within each cartilage sample was determined as an average of three sections. From the second half, water content was determined by calculating the difference between the wet weight and dry weight after freeze-dying the samples for 20 h.

### Statistical analysis

Relation between the known and measured iodine and gadolinium concentrations in the contrast agent mixtures was studied using Pearson’s correlation. As our study had relatively small number of samples and since Shapiro-Wilk test showed that the data was not from a normally distributed population, a nonparametric test was chosen. Therefore, significance of the differences in parameter values between the intact and injured samples were calculated utilizing Wilcoxon signed-rank test. The statistical significance of dependence between the contrast agent partitions in superficial cartilage layer (20% of the cartilage thickness) and optical density was determined. All statistical analyses were conducted using SPSS software (v. 24.0 SPSS Inc., IBM Company, USA). *P* < 0.05 was the limit for statistical significance.

## Results

**Figure 1:**
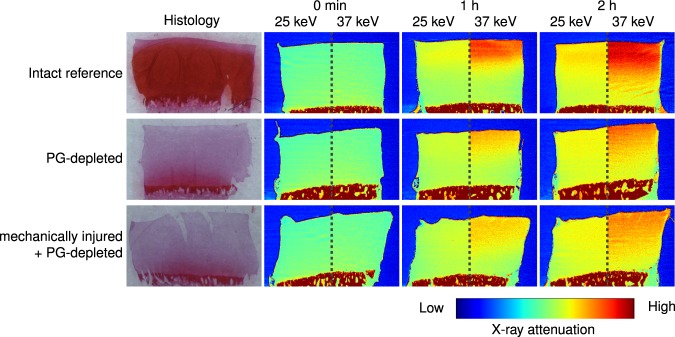
Safranin-O stained histological section and synchrotron microCT images (average of 21 consecutive 6.5 µm thick slices) of representative intact, proteoglycan (PG) depleted, and mechanically injured + PG-depleted samples, as acquired with both photon energies (25 keV and 37 keV energies) prior to the contrast agent immersion and 1 and 2 h after the immersion. These synchrotron microCT images highlight the changes in X-ray attenuation at two photon energies during diffusion of contrast agent into articular cartilage. Based on these images, the depth-wise partitions of gadoteridol and CA4+ is solved using the Beer-Lambert law and Bragg’s rule.

**Figure 2:**
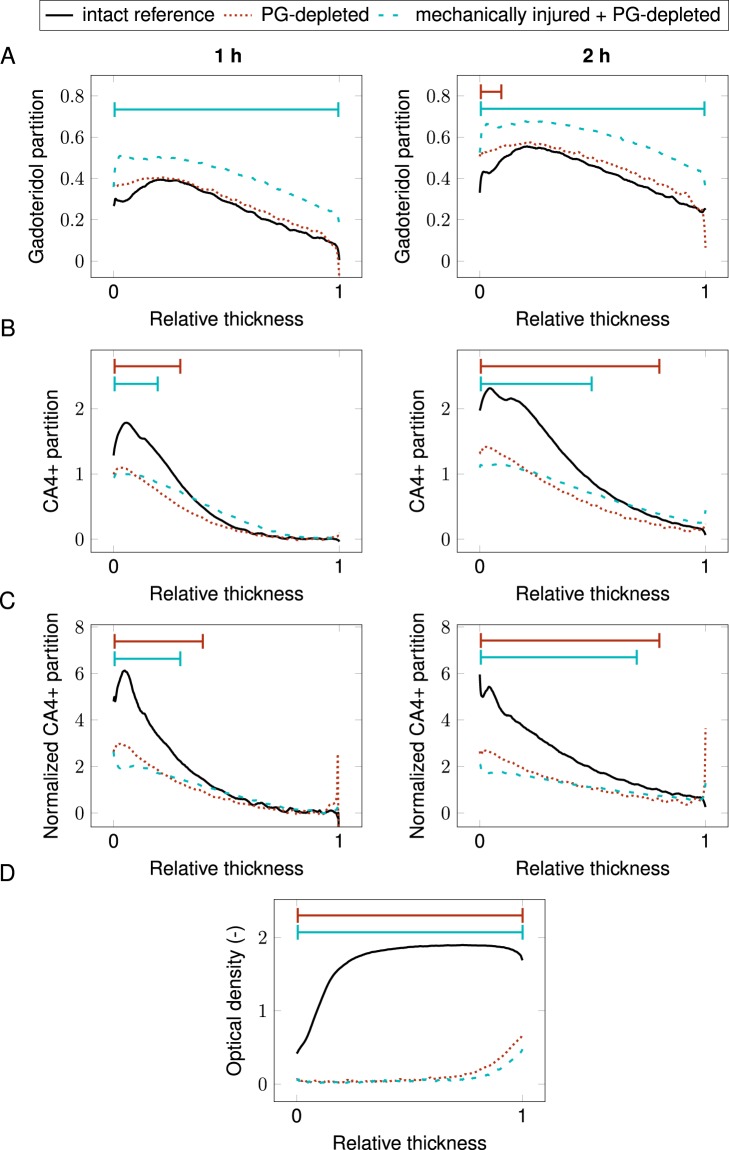
Mean depth-wise partitions of (**A**) gadoteridol, (**B**) CA4+, and (**C**) normalized CA4+ in cartilage after 1 h and 2 h immersion in contrast agent mixture. (**D**) optical density profile through cartilage thickness.  represents the statistically significant (*P* < 0.05) difference between intact and damaged samples. 0 denotes the cartilage surface and 1 cartilage-bone interface.

**Figure 3:**
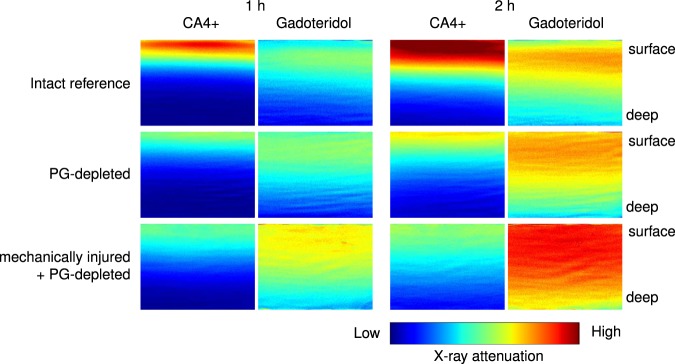
CA4+ and gadoteridol distribution within cartilage (cartilage surface on the top edge of the image and cartilage-bone interface on the bottom edge) at 1 and 2 h after contrast agent immersion for intact, proteoglycan (PG) depleted, and mechanically injured + PG-depleted samples. CA4+ distributes into cartilage proportionally to PG content while gadoteridol distributes related to water content and steric hindrance of the tissue.

Measured iodine and gadolinium concentrations in the contrast agent mixtures correlated linearly with the known concentrations (*R*^2^ = 0.99, *P* < 0.001, Table 1), the average error being 2.89%. Synchrotron microCT images, obtained with the two energies, showing changes in X-ray attenuation during contrast agent diffusion together with corresponding histological images are presented in Fig. 1. The uptake of non-ionic gadoteridol into mechanically injured + PG-depleted samples was significantly higher than into intact reference samples at both imaging time points (*P* < 0.008, Fig. 2). No significant difference in gadoteridol uptake was observed between the intact reference and PG-depleted samples. The uptake of cationic agent (CA4+), as a function of cartilage depth, was significantly higher in the intact samples than in damaged samples (Fig. 2B). After normalization of the CA4+ partition with the gadoteridol partition, cartilage degradation and injury were more effectively detected. The detection of these features was significantly improved, especially, for the mechanically injured samples, as indicated by the statistically significant differences present in the deeper cartilage depth as shown in Fig. 2. No statistically significant changes in water contents between the sample groups were observed in water content analysis. Concentration maps of CA4+ and gadoteridol are shown in Fig. 3. The PG content (i.e., OD of Safranin-O stained sections) was significantly lower in PG-depleted (*P* = 0.008) and mechanically injured + PG-depleted samples (*P* = 0.008) than in intact samples (Figs 1 and 2D). Correlations between OD and CA4+ partition values were determined for the superficial cartilage layer (20% of the cartilage thickness); the obtained Spearman’s rhos (*P* < 0.001) for non-normalized CA4+ partitions after 1 and 2 h were *ρ* = 0.683 and *ρ* = 0.738, respectively. For normalized CA4+ partitions the spearman’s rhos after 1 and 2 h were *ρ* = 0.734 and *ρ* = 0.662.

**Table 1:** Known and measured iodine and gadolinium concentrations in the contrast agent mixtures.

	Known concentration (mg I/ml)/(mg Gd/ml)	Measured concentration (mg I/ml)/(mg Gd/ml)	Error (%)
Mixture 1	20/5	19.64/5.14	1.8/2.8
Mixture 2	10/10	10.09/9.92	0.9/0.8
Mixture 3	5/20	4.88/19.61	2.4/2.0
Mixture 4	3/3	2.84/3.22	5.3/7.3

## Discussion

In this synchrotron-based microCT study, the QDECT technique is evaluated along with a dual contrast agent solution consisting of a mixture of the cationic, iodinated contrast agent (CA4+) and the non-ionic, gadolinium-based contrast agent (gadoteridol). The application of cationic contrast agents for imaging soon after the contrast agent administration is challenging as the diffusion of cationic contrast agent into cartilage is governed by the composition and structure of cartilage extracellular matrix. In degenerated cartilage, reduced PG content decreases the diffusion of the cationic agent while weakened steric hindrance and elevated water content increase agent diffusion resulting in opposite effects on the diffusion^43^. In this study, we demonstrate that by using QDECT, with a mixture of cationic and non-ionic contrast agents, cartilage PG content and changes in water content and steric hindrance can be evaluated simultaneously. In addition, mechanical impact was found to increase the uptake of non-charged gadoteridol into cartilage. Furthermore, the use of monochromatic X-ray energies eliminates the problems arising from the use of continuous X-ray spectrum.

The results show, known CA4+ and gadoteridol concentrations in the contrast agent mixtures correlate linearly with the measured concentrations *(R*^2^ = 0.99, *P* < 0.001), indicating that CA4+ and gadoteridol concentrations in aqueous solution can be measured accurately using QDECT. Depth-wise contrast agent partition profiles, acquired with synchrotron microCT (Fig. 2), illustrate the highest gadoteridol uptake is observed with mechanically injured, PG-depleted samples. This increase in gadoteridol uptake is attributed to reduced articular cartilage steric hindrance due to mechanical impact^28,32^ affording greater porosity and increased surface area, owing to the cracks on the cartilage surface.

The uptake of CA4+ was found to be significantly higher in intact samples than in injured samples. This result is consistent with previous observations^22,23,31,44^ that showed that the cationic CA4+ uptake is proportionally related to the cartilage PG content^14,22^, and intact cartilage has higher PG content compared to injured samples. When comparing the PG-depleted samples with the mechanically injured + PG-depleted samples, the latter group shows slightly higher CA4+ uptake than the PG-depleted samples. This is likely due to reduced steric hindrance caused by the mechanical damage as water content showed no significant changes in water content measurements. This result indicates that reduced steric hindrance affects the diagnostic potential of CA4+.

At early time points of diffusion, CA4+ is unable to distinguish intact (e.g., high PG content) from degenerated cartilage (e.g., mechanical injured), as similar uptake ratios are observed^43^. We hypothesized that the sensitivity of cationic CA4+ in early time points could be improved by normalizing the CA4+ partition with that of gadoteridol, a non-ionic contrast agent. The present results show that normalizing the CA4+ partition with that of gadoteridol increases the capability of CA4+ to differentiate injured and intact tissue. This normalization procedure improves the diagnostic sensitivity of CA4+ to detect PG content, however, only moderately since the enzymatic degradation and mechanical impact had no statistical significant effect on tissue water content^32,36^. In arthritic cartilage, the water content is increased by approximately 10%^45,46^. Thus, the normalization of CA4+ attenuation can increase its diagnostic sensitivity of arthritic cartilage.

To obtain optimal results, the acquisitions of the imaging data are conducted as consecutively as possible using the two energies. At early time points after contrast agent immersion, the diffusion rate is at its highest and the depth-wise partition is constantly changing. Thus, conducting these acquisitions as consecutively as possible is of utmost importance. The time difference between the starting points of 25 keV and 37 keV acquisitions was approximately 5 min 20 s, and the acquisition took approximately 128 seconds. During this time, diffusion continues to occur within the cartilage. Thus, as the obtained attenuation profiles with two energies are not obtained exactly at the same time point, the X-ray attenuation profiles represent an average value over the scan time rather than a precise value. Nevertheless, these issues minimally impacted the results or conclusions as the imaging settings were similar for all the samples and the samples were of similar thickness.

Compared with MRI, the drawback of QDECT is the application of ionizing radiation. However, the patient doses used in routine knee imaging are small (27–48 μSv effective dose) and even lower (13 μSv effective dose) with modern orthopedic cone beam CT scanners^47^. A further limitation of the current CECT technique is the requirement of two scans (arthrographic scan and delayed scan). In a clinical setting, performing two scans is time consuming doubling the radiation dose and being logistically challenging. Fortunately, as dual-energy CT scanners have become more widespread, imaging with two energies is now conducted simultaneously, supporting the application of the QDECT.

In this paper, we describe a proof-of-principle approach to study the potential of QDECT for cartilage diagnostics at clinically feasible imaging time points. Future studies will focus on minimizing the problems arising from the continuous X-ray energy spectra applied in clinical devices and exploring the technique in a true clinical setting for *in vivo* diagnostics. Additionally, as photon counting detector (PCD) systems with energy discrimination capabilities have evolved rapidly during the last 10 years, the potential of QDECT together with PCD utilizing two energy thresholds should be examined.

In conclusion, QDECT allows simultaneous and quantitative evaluation of cationic and non-ionic contrast agent uptake within cartilage at early imaging time points. The shortcoming related to CA4+ diffusion is minimized by normalizing its uptake with that of a non-ionic contrast agent. Further, by utilizing monochromatic energies the problems related to continuous X-ray spectra (mainly beam hardening) are avoided. The present dual contrast method provides valuable information on early tissue changes in cartilage related to PTOA. Specifically, the uptake of cationic agent (CA4+) was significantly lower (*P* < 0.05) in both injured sample group compared with the intact samples, confirming reduced PG content. Moreover, after normalizing the CA4+ partition with that of gadoteridol (e.g., reducing the effect of reduced steric hindrance and increased water content on contrast agent diffusion), the detection of cartilage degradation and injury was significantly improved. Although, the number of samples was relatively low (*n* = 11/group, resulting in total of *N* = 33 samples), the results were found statistically significant with the current number of samples. Potentially, QDECT will allow enhanced diagnostics of the disease, improved planning of surgical treatment options, and better strategies for preventing OA after a traumatic injury.

## Data Availability

All data are available from the corresponding author upon reasonable request.
